# Rapid Detection of Neutrophil Oxidative Burst Capacity is Predictive of Whole Blood Cytokine Responses

**DOI:** 10.1371/journal.pone.0146105

**Published:** 2015-12-30

**Authors:** Philip J. Vernon, Leasha J. Schaub, Jurandir J. Dallelucca, Anthony E. Pusateri, Forest R. Sheppard

**Affiliations:** 1 Naval Medical Research Unit San Antonio, JBSA-Ft. Sam Houston, Texas, United States of America; 2 59^th^ Medical Wing, US Air Force, JBSA-Ft. Sam Houston, Texas, United States of America; 3 US Army Medical Research and Materiel Command, Ft. Detrick, Maryland, United States of America; Seattle Biomedical Research Institute, UNITED STATES

## Abstract

**Background:**

Maladaptive immune responses, particularly cytokine and chemokine-driven, are a significant contributor to the deleterious inflammation present in many types of injury and infection. Widely available applications to rapidly assess individual inflammatory capacity could permit identification of patients at risk for exacerbated immune responses and guide therapy. Here we evaluate neutrophil oxidative burst (NOX) capacity measured by plate reader to immuno-type Rhesus Macaques as an acute strategy to rapidly detect inflammatory capacity and predict maladaptive immune responses as assayed by cytokine array.

**Methods:**

Whole blood was collected from anesthetized Rhesus Macaques (n = 25) and analyzed for plasma cytokine secretion (23-plex Luminex assay) and NOX capacity. For cytokine secretion, paired samples were either unstimulated or *ex-vivo* lipopolysaccharide (LPS)-stimulated (100μg/mL/24h). NOX capacity was measured in dihydrorhodamine-123 loaded samples following phorbol 12-myristate 13-acetate (PMA)/ionomycin treatment. Pearson’s test was utilized to correlate NOX capacity with cytokine secretion, p<0.05 considered significant.

**Results:**

LPS stimulation induced secretion of the inflammatory molecules G-CSF, IL-1β, IL-1RA, IL-6, IL-10, IL-12/23(p40), IL-18, MIP-1α, MIP-1β, and TNFα. Although values were variable, several cytokines correlated with NOX capacity, p-values≤0.0001. Specifically, IL-1β (r = 0.66), IL-6 (r = 0.74), the Th1-polarizing cytokine IL-12/23(p40) (r = 0.78), and TNFα (r = 0.76) were strongly associated with NOX.

**Conclusion:**

NOX capacity correlated with Th1-polarizing cytokine secretion, indicating its ability to rapidly predict inflammatory responses. These data suggest that NOX capacity may quickly identify patients at risk for maladaptive immune responses and who may benefit from immuno-modulatory therapies. Future studies will assess the *in-vivo* predictive value of NOX in animal models of immune-mediated pathologies.

## Introduction

Maladaptive immune responses are the primary mediators of tissue damage and ultimately death in a large number of illnesses [1, 2]. Trauma and hemorrhage, as well as subsequent ischemia-reperfusion injury (IRI) are all examples of clinical scenarios where deleterious inflammation is present [1, 3, 4]. Although these pathologies are complex and heterogeneous in nature, outcomes can be equally challenging to predict due to the confluence of multiple factors encompassing components of both the innate and adaptive arms of the immune system [5–8]. One of the primary challenges for determining susceptibility to hemorrhagic shock in response to trauma is the wide-ranging variability between individual patient inflammatory responses [5, 9]. Determining the baseline capacity for the immune system to respond to a stimulus *ex-vivo* (immuno-typing) has been posited as a vehicle to both predict susceptibility to shock and to inform clinical decision-making [10]. A readily deployable technique for rapidly assessing inflammatory capacity across patients utilizing widely-available hospital technology would be ideal.

Adaptive lymphocytes including both CD4^+^ helper T cells (Th) and CD8^+^ cytotoxic lymphocytes (CTLs) become activated in response to injury. These cells mediate damaging and systemic inflammatory effects with the potential of culminating in multiple organ failure 24–48 hours post-injury [11, 12]. One of the primary subsets of effector cells attributed with pathological activity not only in response to trauma but also in autoimmune disorders, gastrointestinal inflammatory diseases, and transplant rejection are T helper type 1 or Th1 cells [13, 14]. Naïve CD4^+^ T cells polarize towards a Th1 phenotype in response to the secretion of IL-12 from an antigen-presenting cell (APC) at the immunologic synapse [15]. Th1 cells are characterized by the transcriptional activities of T-bet and the production and active secretion of the cytokine IFNγ[16, 17]. IFNγ mediates its effects at the site of injury by increasing antigen presentation on surrounding cells, enhancing natural killer (NK) cell function, and further maturing and activating tissue-resident APCs such as macrophages and dendritic cells (DCs) [18, 19].

Immediately upon cell damage or tissue injury, soluble inflammatory factors such as damage associated molecular pattern (DAMP) molecules and chemokines are released into the extracellular milieu where they recruit and activate innate lymphocytes [20, 21]. For example, in the event of infection or injury, circulating neutrophil granulocytes extravasate into the tissue from peripheral blood in response to a gradient of the chemokine IL-8 where they promote reparative wound healing and fulfill an antimicrobial role [22, 23]. Following injury, neutrophils can be identified pervasively throughout tissue engaged in phagocytosis, oxidative bursts, degranulation, and the more recently described neutrophil extracellular traps (NETs) [24–26]. Neutrophil oxidative bursts (NOX), while being effective in the disruption of infectious organisms, are particularly damaging during sterile injury and trauma [27]. Neutrophil-derived reactive oxygen species (ROS) significantly contribute to both initial acute-phase injury and subsequent systemic complications [10, 28]. The inhibition of nicotinamide adenine dinucleotide phosphate (NADPH) oxidase in neutrophils has been shown to mitigate injury in a number of studies [29–31]. Furthermore, the criticality of neutrophils in regulating T cell responses has also been demonstrated [32]. In transgenic mice strains lacking CD8^+^ T cells, neutrophil infiltrate is significantly attenuated, indicating that these innate and adaptive responses are integrated [32]. The diminished infiltrate also suggests the possibility that innate neutrophil activity may be regulated by adaptive T cell responses, providing an opportunity for clinical intervention in trauma patients deemed high-risk for septic or hemorrhagic shock and IRI.

Neutrophil oxidative burst capacities assayed by flow cytometry are routinely used to diagnose congenital Chronic Granulomatous Disease (CGD) in hospitals using freshly collected whole blood from high-risk individuals [33]. CGD is caused by a mutation in a subunit of membrane-bound NADPH oxidase-2 rendering neutrophil oxidative bursts impossible [34, 35]. NADPH oxidase generates ROS effective in the lysing of pathogens, therefore CGD patients experience chronic and recurring infections [35]. Patient samples are treated with phorbol 12-myristate 13-acetate (PMA), a potent protein kinase C (PKC) agonist, to induce oxidative bursts and thus the production of ROS. Following this stimulation, samples are incubated with the fluorogenic molecule dihydrorhodamine-123 (DHR-123). DHR-123 is oxidized into fluorescent rhodamine-123 (R-123) in the presence of ROS which is measured via flow cytometer in the FITC (488nm) channel [36]. An absence of detectable R-123 in comparison to a healthy donor control is indicative of a lack of NADPH oxidase activity and indicative of the CDG mutation.

Importantly, studies have demonstrated NOX capacity measured pre-operatively is predictive of the development of post-operative pneumonia. The predictive nature of such a technique would be extremely useful in populations at high risk for trauma-related injuries such as military personnel in combat scenarios and civilians routinely exposed to heavy machinery in industrial and agricultural settings [10]. Therefore, we postulate that baseline NOX capacity determination may predict inflammatory responses and thereby facilitate patient-individualized therapy. This would ensure intervention is appropriate both in application and magnitude to avoid deleterious inflammatory consequences. In this study, we have developed a microplate-based NOX capacity assay derived from the clinically-available flow cytometric assay for CGD diagnosis utilizing PMA. We have optimized and evaluated the ability of this readily deployable assay to predict the profile and extent of cytokine secretion in response to *ex-vivo* whole blood treatment with a naturally occurring and immunologically relevant stimulus, lipopolysaccharide (LPS).

## Materials and Methods

### Rhesus Macaque Use and IACUC Compliance

Rhesus macaques (*Macaca mullata*) were housed at the Tri-Services Research Laboratory (TSRL) at Joint Base San Antonio (JBSA)-Fort Sam Houston, TX. The study protocol was approved by the Institutional Animal Care and Use Committee (IACUC) at the 711th Human Performance Wing, JBSA-Fort Sam Houston, and conducted in accordance with the Guide for the Care and Use of Laboratory Animals, Institute of Laboratory Animals Resources, National Research Council, National Academy Press, 2011. All procedures were performed in facilities accredited by the Association for Assessment and Accreditation for Laboratory Animal Care International (AAALAC). The animals were pair housed in compliance with the Secretary of the Navy Instruction (SECNAVINST) 3900.38C regulations with *ad libitum* access to environmental enrichment, feed and water and exposed to 12hr/12hr light/dark cycles. Specific environmental enrichment included toys (rings, balls and kongs), edible treats, access to television and cages equipped with mirrors and swings. No animals were sacrificed for this study.

### Rhesus Macaque Blood Draw Procedure

25 Rhesus macaques were sedated with 2mg/kg of 50mg/ml 1:1 tiletamine/zolazepam intramuscularly (Fort Dodge Animal Health, Fort Dodge, IA, USA) and baseline whole blood collected via femoral blind stick in vacutainers using EDTA as an anti-coagulant during routine quarantine procedures (BD Biosciences, San Jose, CA, USA). Samples were assigned a numerical value for blinding purposes and paired for use in both NOX capacity and whole blood stimulation assays.

### Rhesus Macaque Whole Blood Cell Quantification

Complete blood count (CBC) was determined via HemaTrue veterinary hematology analyzer (HESKA, Loveland, CO, USA). Blood was kept at room temperature and never stored for longer than 16 hours prior to analysis.

### Whole Blood Stimulation

A total of 250μl of whole Rhesus Macaque blood was stimulated with 10, 100 and 1000μg/ml of LPS (Sigma-Aldrich, St. Louis, MO, USA) to determine the optimal stimulating concentration. 100μg/ml was selected and used for whole blood stimulation assays. Samples were incubated at 37°C with 5% CO_2_ for 24 hours. Blood was centrifuged at 1800xg for 15 minutes. Plasma supernatant was then analyzed for the presence of cytokines, chemokines and inflammatory growth factors.

### Multiplex Cytokine Analysis

Cytokine concentrations contained within the plasma supernatants of LPS-stimulated whole blood were quantified using the Bio-plex 200 Systems Luminex plate reader (Bio-Rad Laboratories, Hercules, CA, USA). A 23-plex non-human primate panel was evaluated using the Milliplex Non-Human Primate Cytokine Magnetic Bead Panel-Premixed Immunology Multiplex Assay (EMD Millipore, Billerica, MA, USA) according to the manufacturer’s instructions with washing steps being completed in the ELx405 Select Deep Well Washer magnetic plate washer (BioTek, Winooski, VT, USA). The panel was comprised of the following analytes: granulocyte-colony stimulating factor (G-CSF), granulocyte-macrophage-colony stimulating factor (GM-CSF), interferon gamma (IFNγ), interleukin 1β (IL-1β), IL-1 receptor agonist (IL-1RA), IL-2, IL-4, IL-5, IL-6, IL-8, IL-10, IL-12/23(p40), IL-13, IL-15, IL-17A, IL-18, macrophage chemotactic protein-1 (MCP-1 or CCL2), macrophage inflammatory protein alpha (MIP-1α or CCL3), macrophage inflammatory protein beta (MIP-1β or CCL4), soluble CD40 ligand (sCD40L), transforming growth factor alpha (TGFα), tumor necrosis factor alpha (TNFα), and vascular endothelial growth factor (VEGF). Results were analyzed using Bio-Plex Manager Software V 6.1 (Bio-Rad Laboratories, Hercules, CA, USA).

### Cell lines

HL-60 human neutrophil-like cells used in neutrophil oxidative burst assay development and optimization were acquired from American Type Culture Collection (ATCC, Manassas, VA, USA).

### Analysis of Neutrophil Oxidative Burst Capacity

To determine neutrophil oxidative burst capacity, DHR-123 conversion into the fluorophore rhodamine-123 (R-123) was detected at 488nm using the Synergy H1 Hybrid Reader microplate reader (BioTek, Winooski, VT, USA) and the FACSAria III flow cytometer and cell sorter (BD Biosciences, San Jose, CA, USA). Microplate data were analyzed by Gen5 Data Analysis Software (BioTek, Winooski, VT, USA). Flow cytometry data were analyzed by FlowJo V.10 (Tree Star, Inc., Ashland, OR, USA).

### Optimization of Microplate-based Neutrophil Oxidative Burst Capacity Assay

The following microplate reader parameters were evaluated and optimized to discern intra-assay conditions rendering the highest possible fluorescent signal detectable in the samples with the greatest magnitude of difference between samples and negative controls in HL-60 cells: dose titrations of 1–2μg/ml of DHR-123, dose titrations of 2.5-10ng/ml of PMA alone or a combination reagent containing both PMA and the calcium ionophore, ionomycin, clear-bottom versus black-bottom 96 well plate formats, and detecting fluorescence at 488nm from detectors both below (bottom read) or above (top read) the samples.

### Rhesus Macaque Neutrophil Stimulation

Volumes of Rhesus Macaque whole blood containing a total of 5x10^5^ neutrophils, calculated from the CBC value determined as described above, were suspended in 500μl of phosphate buffered saline (PBS) for flow cytometric analysis and 200μl for microplate reader analysis. Sample volumes were calculated based on concentrations of granulocytes determined via complete blood count. Cells were then stimulated with Leukocyte Activation Cocktail (10μg/ml of phorbal 12-myristate 13-acetate, 250μg/ml of ionomycin, and 0.5mg/ml Brefeldin A) (BD Biosciences, San Jose, CA, USA) for 30 minutes at 37°C in 5% CO_2_. During the final 5 minutes of incubation, 1μg/ml of DHR-123 (Sigma-Aldrich, St. Louis, MO, USA) was added. Cells were then immediately placed on ice to stop all reactions.

### Statistical Analysis

For intra- and inter-assay analysis, statistical significance was determined using a two-tailed Student’s T Test with p-values of p<0.05 being designated as significant. NOX microplate reader-based assay effectiveness was determined by comparing generated values with those obtained from the above mentioned flow cytometry method. Correlation coefficient values (r) for the comparison of NOX capacity with cytokine secretion were determined using the Pearson’s Test with p-values of p<0.05 being designated as significant and p-values of p≤0.0001 being designated as significant for predictive analysis. Outliers identified in NOX capacity assays were defined as data points measured to be three standard deviations outside of the mean. Outliers identified during multiplex analysis were defined as data points measured to be five standard deviations outside of the mean. All statistical analysis was performed using GraphPad Prism 6 (GraphPad Software, Inc., La Jolla, CA, USA).

## Results

### Characterization of the cytokine response of whole blood stimulated *ex-vivo*

**Fig 1:**
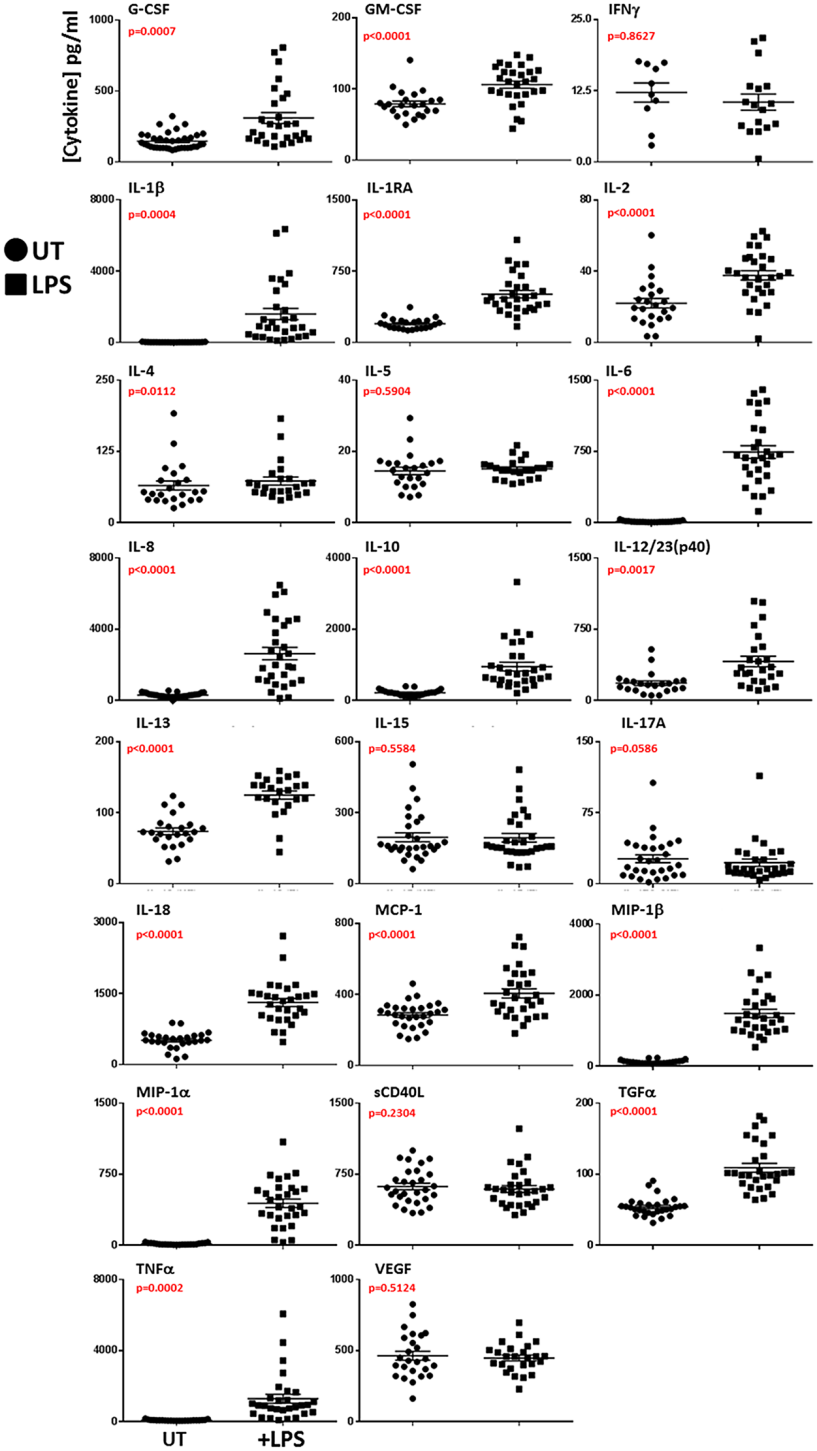
Cytokine secretion in response to LPS stimulation in Rhesus Macaque whole blood. Scatter plots depicting the individual responses (pg/ml) in each of the 23 cytokines measured in Rhesus Macaque whole blood stimulated with 100ng/ml LPS for 24 hours or not (UT). Cytokines were assayed by multiplex analysis of plasma supernatant.

**Fig 2:**
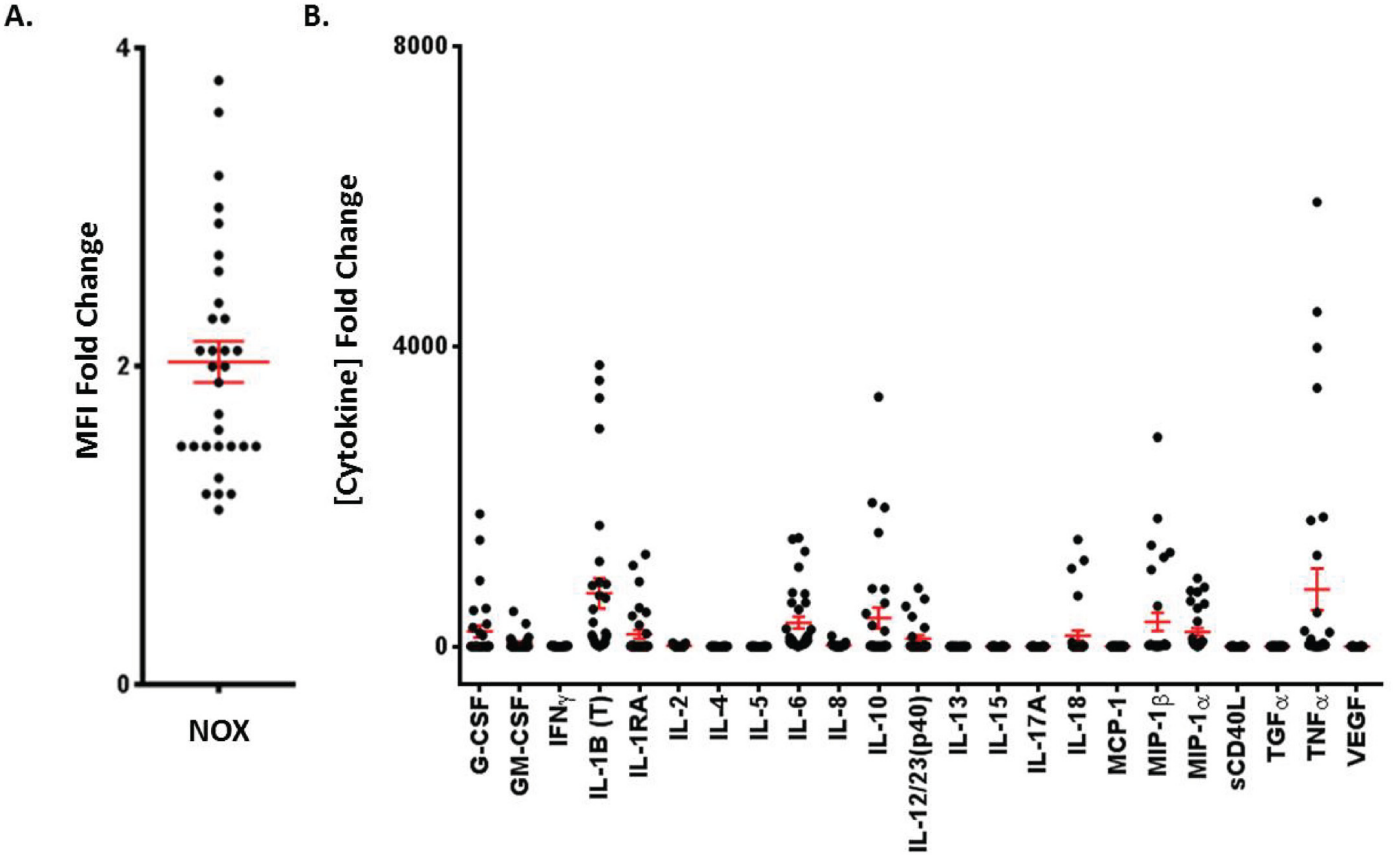
Immuno-typing Non-human primate baseline neutrophil oxidative burst capacity and whole blood cytokine production in response to LPS stimulation. (A) 5 x 10^5^ Rhesus Macaque neutrophils were stimulated or not with 10ng/ml PMA and 1μg/ml ionomycin for 30 minutes. During the final 5 minutes of incubation, cells were loaded with 1μg/ml DHR-123 and analyzed by microplate reader at 488nm. The fold changes in mean fluorescence intensity (MFI) between stimulated and unstimulated samples from each non-human primate are depicted. (B) The fold changes of cytokines determined by multiplex analysis between LPS-stimulated and unstimulated whole blood samples from each non-human primate assayed in Fig 1 are depicted.

Fresh Rhesus Macaque whole blood was stimulated with LPS *ex-vivo* and cytokine response profile determined by multiplex analysis. Each cytokine analyzed was significantly elevated (p<0.05) in stimulated samples compared with untreated controls except IFNγ, IL-5, IL-15, IL-17A, sCD40L, and VEGF (Fig 1). The greatest mean fold changes in treated samples were observed for G-CSF, IL-1β, IL-1RA, IL-6, the immuno-regulatory cytokine IL-10, IL-12/23(p40), IL-18, MIP-1α, MIP-1β, and TNFα (p<0.0001) (Figs 1 and 2B).

### Analysis of baseline neutrophil oxidative burst capacity in HL-60 cells

Flow cytometery-based assessment of neutrophil oxidative burst capacity has been extensively validated and its accuracy well-established in clinical settings (S1 Fig), however the cost and logistical issues surrounding flow cytometers can be prohibitive in some scenarios (i.e. combat theater) due to immobility and the necessity for fresh samples. Therefore, we sought to develop a similar PMA/DHR-123 based approach using a more cost-effective and deployable instrument: a microplate reader with fluorescent detection capacity utilizing HL-60 human neutrophil-like cells. As depicted in S2 Fig, the optimal conditions for a microplate reader-based NOX capacity assay were determined to be 1μg/ml DHR-123, 10ng/ml PMA and 1μg/ml ionomycin detected using a top read in black-bottom plates.

### Analysis of baseline neutrophil oxidative burst capacity in whole blood and comparison to flow cytometric method

Next, paired samples of Rhesus Macaque whole blood was assayed for NOX capacity by both microplate reader and flow cytometer. The mean fold change was 2.081 with a standard deviation of 1.03 in our microplate reader assay (Fig 2A). Similar to observed whole blood cytokine secretion in response to LPS, baseline NOX capacities were variable amongst individuals within the cohort (Fig 2A and 2B). Furthermore, a direct comparison between NOX capacity fold changes obtained by the microplate reader and flow cytometry was performed (S3 Fig). The microplate reader assay performed as effectively as the flow cytometry assay (p = 0.9793), and no significant changes in sensitivity or variation were observed between assays. Furthermore, both assays detected equivalent NOX capacities on an individual animal basis.

### Baseline neutrophil oxidative burst capacity is predictive of *ex-vivo* peripheral whole blood production of IL-1β, IL-6, TNFα, and the Th1-polarizing cytokine, IL-12/23(p40)

Paired data obtained from both whole blood stimulation and NOX capacity assays were then compared. The Pearson’s Test for correlations between data sets revealed the ability of the rapid assessment of NOX capacity to predict the production of cytokines, particularly those with integral roles in shaping downstream maladaptive responses, such Th1-polarizing responses.

**Fig 3:**
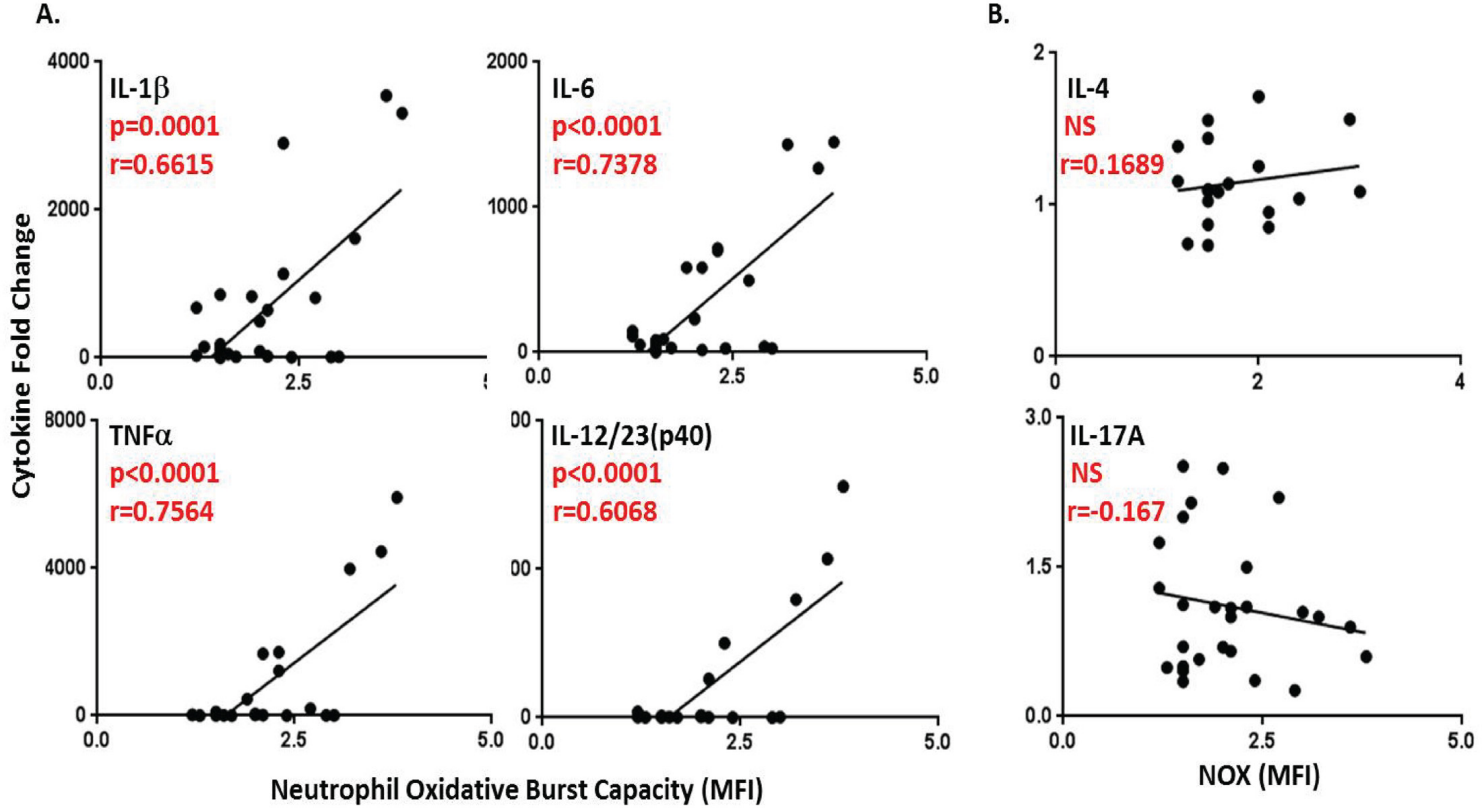
Baseline neutrophil oxidative burst capacity is predictive of *ex-vivo* whole blood production of IL-1β, IL-6, TNFα, and the Th1-polarizing cytokine, IL-12/23(p40). (A) Scatter plots of cytokine fold change (y-axis) and neutrophil oxidative burst fold change in MFI (x-axis) that were determined to have statistically significant correlative coefficient (r) values (p≤0.0001) by Pearson’s Test. (B) Scatter plots of cytokine fold change and neutrophil oxidative burst capacity fold change for two cytokines found to be not statistically significant: IL-4 (Th2 cytokine), IL-17 (Th17 cytokine).

Neutrophil oxidative burst capacity was determined to be positively correlated with the secretion of the cytokines G-CSF, GM-CSF, IL-1β, IL-1RA, IL-2, IL-6, IL-8, IL-12/23(p40), MIP-1α, and TNFα (p<0.05) (Table 1). For the purpose of determining the predictive ability of NOX capacity with regard to cytokine production, a much more stringent statistical threshold of p≤0.0001 was used. NOX capacity measured rapidly in fresh blood could predict the production of IL-1β (r = 0.6615, R^2^ = 0.4356), IL-6 (r = 0.7378, R^2^ = 0.5443) TNFα (r = 0.7564, R^2^ = 0.5722) and the Th1-polarizing cytokine IL-12/23(p40) (r = 0.7789, R^2^ = 0.6068) during a 24 hour *ex-vivo* stimulation with a high degree of accuracy (Fig 3A). Conversely, the Th2-associated cytokine IL-4 and the Th17-associated cytokine IL-17A were not correlated with NOX capacity with r values of 0.1689 and -0.167 and R^2^ values of 0.02853 and 0.02789, respectively (Fig 3B). These data suggest that in addition to the ability of NOX capacity to predict the extent of production of the broad pro-inflammatory markers IL1β, IL-6 and TNFα, which are generally present in elevated quantities during most tissue injury, it is also predictive of the specifically Th1-polarizing cytokine, IL-12/23(p40).

**Table 1:** Correlation values between neutrophil oxidative burst capacity and 23 cytokines, chemokines and growth factors.

Cytokine	r	R^2^	p-value	
G-CSF	0.4338	0.1882	0.0238	*
GM-CSF	0.4862	0.2364	0.0118	*
IFNγ	-0.1452	0.0211	0.5781	NS
IL-1β	0.6615	0.4376	0.0001	***
IL-1RA	0.4273	0.1826	0.0262	*
IL-2	0.4250	0.1807	0.0304	*
IL-4	0.1689	0.0285	0.4894	NS
IL-5	0.2256	0.0509	0.3530	NS
IL-6	0.7378	0.5443	<0.0001	****
IL-8	0.4720	0.2228	0.0S129	*
IL-10	0.2739	0.0750	0.1668	NS
IL-12/23(p40)	0.7789	0.6068	<0.0001	****
IL-13	-0.4001	0.1601	0.0895	NS
IL-15	0.2115	0.0448	0.2895	NS
IL-17A	-0.1670	0.0279	0.4051	NS
IL-18	0.3517	0.1237	0.0720	NS
MCP-1	-0.0060	3.620e-05	0.9762	NS
MIP-1β	0.3050	0.0930	0.1219	NS
MIP-1α	0.4809	0.2312	0.0111	*
sCD40L	0.0469	0.0022	0.8165	NS
TGFα	0.3436	0.1108	0.0793	NS
TNFα	0.7564	0.5722	<0.0001	****
VEGF	-0.1948	0.0379	0.3618	NS

Table depicting the individual correlative values determined using Pearson’s Test between each of the 23 cytokines, chemokines and growth factors analyzed and neutrophil oxidative burst capacity.

* (p<0.05),

** (p<0.01),

*** (p<0.001) and

**** (p<0.0001).

## Discussion

The ability to rapidly predict potentially deleterious adaptive immune responses would be an invaluable clinical asset in a variety of inflammatory pathologies ranging from hemorrhagic and septic shock resulting from trauma and infection, respectively, to graft versus host and autoimmune diseases. In addition to buying precious time in an emergency medicine environment by anticipating the occurrence and severity of harmful inflammation early, it would enable individuals who are at a high risk for traumatic injury, such as military personnel or individuals exposed to heavy machinery, to be classified according to immuno-type well before adverse medical events occur. This could significantly improve triage and intervention practices in response to trauma.

We have demonstrated that the clinically-available flow cytometric assay for neutrophil oxidative burst capacity as a diagnostic for Chronic Granulomatous Disease can be transitioned to a more readily deployable and cost-effective microplate reader-based format. The microplate assay also maintains effective performance very comparable to established flow cytometric methodology. This rapid (30 minute incubation) assay is ideally suited for an emergency medicine environment where the occurrence of traumatic injury necessitates the implementation of time-saving techniques. Importantly, these data suggest that NOX capacity is predictive of the production of cytokines from peripheral blood leukocytes integral to the induction of Th1 responses (p≤0.0001) such as IL-1β, IL-6, TNFα and specifically IL-12/23(p40). It is important to note, however, that NOX capacity was assayed in whole blood normalized to neutrophil count as opposed to purified isolates. Therefore, additional cell populations (i.e. undifferentiated monocytes and circulating macrophages) are presumably contributing to detected ROS levels *ex-vivo* despite being present in much lower frequencies.

Th1 cell infiltration into tissue damaged in response to trauma, hemorrhage and/or IRI is known to exacerbate pathologic inflammation primarily through the secretion of IFNγ [37]. IFNγ induces the enhanced expression of major histocompatibility complex (MHC) class I and class II molecules in addition to maturing tissue-resident APCs and enhancing the cytolytic functions of CD8^+^ T cells and NK cells [37, 38]. This results in additional host cell death and can lead to systemic tissue and organ damage and eventually death [38]. The ability to determine a patient’s risk for a Th1 response via NOX capacity would provide the opportunity to intervene therapeutically thus preventing or blunting downstream immune-mediated pathology.

Future studies will utilize human whole blood samples derived from healthy donors, however, we conducted this study using Rhesus Macaques due to their superior ability to model human inflammatory responses and because samples were readily available during routine facility quarantine periods. Additionally, these animals are enrolled in a poly-trauma model development protocol to occur in the future, thereby allowing the possibility of correlative conclusions in planned experiments. These studies will examine results from the data presented here and validate any correlative results with adaptive immune responses and physiologic outcomes in response to trauma observed at the conclusion of the protocol. While these data demonstrate the statistically significant positive correlation between neutrophil oxidative burst capacity and the production of Th1-polarizing cytokines in response to LPS stimulation *ex-vivo*, future studies are critical to determining if these increased systemic cytokines culminate in measurable increases in pathologic Th1 responses *in-vivo*. Specifically, the ability to identify CD3^+^CD4^+^ T cell infiltrate with upregulated IL-2 receptor expression (CD25) in damaged tissue biopsies after trauma/hemorrhage and correlate these cell infiltrate frequencies with NOX capacities recorded in our immuno-typing database is crucial to validating our approach. Furthermore, the detection of IFNγ secretion from these cells after they have been primed in the lymph node by IL-12-secreting APCs is also critical. Furthermore, physiologic outcomes (histology, survival, etc.) must also be correlated with increases in NOX capacity (and therefore Th1 cytokine secretion). All of these are being presently pursued by our group.

Clinical treatment is trending more and more towards personalized medicine in many ways [39]. This includes genetic screens utilized to tailor drug and biological regimens to individual patients thereby increasing the chances of successful outcomes while limiting risks posed by unnecessary or ineffectual therapies [40]. We posit that the host immune system, while being a mediator in much pathology, represents the ultimate example of personalized medicine. Thus, immuno-typing patients using predictive parameters, such as NOX capacity, has the potential to provide critical insights into the most effective and individualized treatments in those at high-risk for inflammatory-mediated pathology.

## Disclaimer

The views expressed in this article are those of the author and do not necessarily reflect the official policy or position of the Department of the Navy, Department of Defense nor the U.S. Government. Authors are either a military service member or employees of the U.S. Government. This work was prepared as part of their official duties. Title 17 U.S.C. §105 provides that ‘Copyright protection under this title is not available for any work of the United States Government.’ Title 17 U.S.C. §101 defines a U.S. Government work as a work prepared by a military service member or employee of the U.S. Government as part of that person’s official duties. The study protocol was reviewed and approved by the 711th HPW/RHD JBSA-Fort Sam Houston Institutional Animal Care and Use Committee (IACUC) in compliance with all applicable Federal regulations governing the protection of animals in research.

## Supporting Information

S1 Fig Flow cytometric analysis of neutrophil oxidative burst capacity. (A) Representative flow dot plots showing the increase in FITC positivity indicative of oxidative-burst mediated oxidation of DHR-123 into R-123. Increases in both FITC^+^ and FITC^hi^ gates are observed. (B) Quantified average frequencies and MFI across all samples in both FITC^+^ and FITC^hi^ populations. * (p<0.05).
(PPTX)

S2 Fig Development and optimization of a microplate-based assay for analysis of neutrophil oxidative burst capacity. (A) 5x10^5^ HL-60 human neutrophil-like cells were stimulated or not with PMA/ionomycin for 30 minutes. During the final 5 minutes of stimulation, the media was supplemented with either 1μg/ml (black bars) or 2μg/ml (red bars) DHR-123 and then analyzed for fluorescence at 488nm indicative of the presence of reactive oxygen species. Additional graphs depict comparisons between initial stimulations of PMA or PMA/ionomycin (P/I) and 2.5, 5 or 10ng/ml P/I. (B) Comparisons of optimally stimulated HL-60 cells read from the top or bottom and in black and clear-bottom well plates. * (p<0.05), ** (p<0.01), *** (p<0.001) and **** (p<0.0001).
(PPTX)

S3 Fig Comparison of microplate-based assay and flow cytometry-based assay performance for neutrophil oxidative burst capacity. NOX capacity was measured by flow cytometer and microplate reader in paired samples of Rhesus Macaque whole blood and directly compared. There were no significant differences in assay performance, sensitivity or variability amongst individual samples (p = 0.9793).
(PPTX)
