# Supplementary material for: Rapid Detection of Neutrophil Oxidative Burst Capacity is Predictive of Whole Blood Cytokine Responses
Source: PLoS One. 2015 Dec 30;10(12):e0146105. doi: 10.1371/journal.pone.0146105 (PMC4696850; doi:10.1371/journal.pone.0146105)
Supplement: S1 Fig — (A) Representative flow dot plots showing the increase in FITC positivity indicative of oxidative-burst mediated oxidation of DHR-123 into R-123. Increases in both FITC+ and FITChi gates are observed. (B) Quantified average frequencies and MFI across all samples in both FITC+ and FITChi populations. * (p<0.05). (PPTX) [file pone.0146105.s001.pptx]

## Slide 1
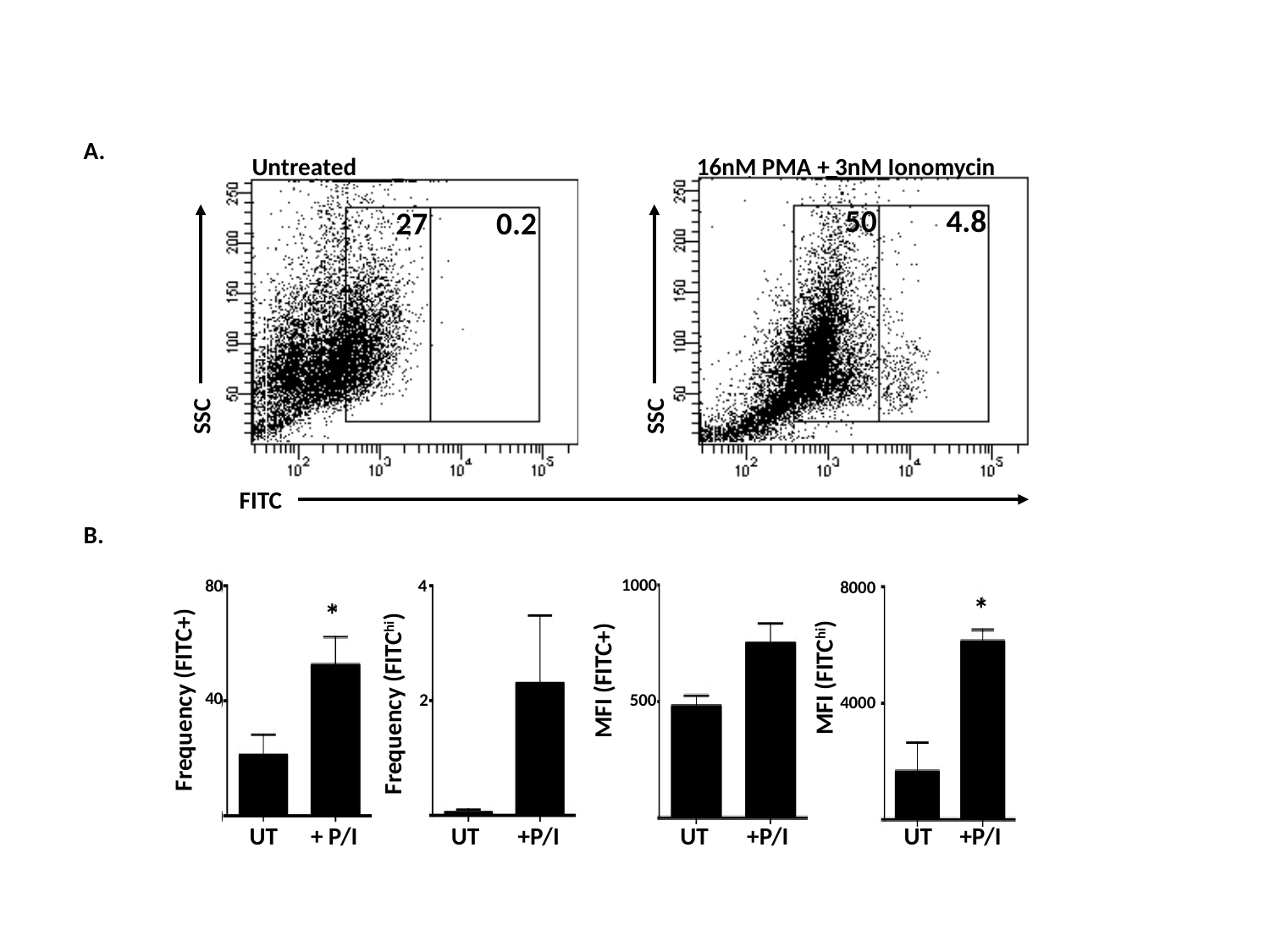

A.
B.
16nM PMA + 3nM Ionomycin
Untreated
4.8
50
27
0.2
SSC
SSC
FITC
1000
80
4
8000
Frequency (FITC+)
*
*
MFI (FITC+)
MFI (FITChi)
Frequency (FITChi)
40
2
500
4000
UT + P/I UT +P/I UT +P/I UT +P/I
