# Supplementary figures and images for: Rapid Detection of Neutrophil Oxidative Burst Capacity is Predictive of Whole Blood Cytokine Responses
Source: PLoS One. 2015 Dec 30;10(12):e0146105. doi: 10.1371/journal.pone.0146105 (PMC4696850; doi:10.1371/journal.pone.0146105)

## Slide 1
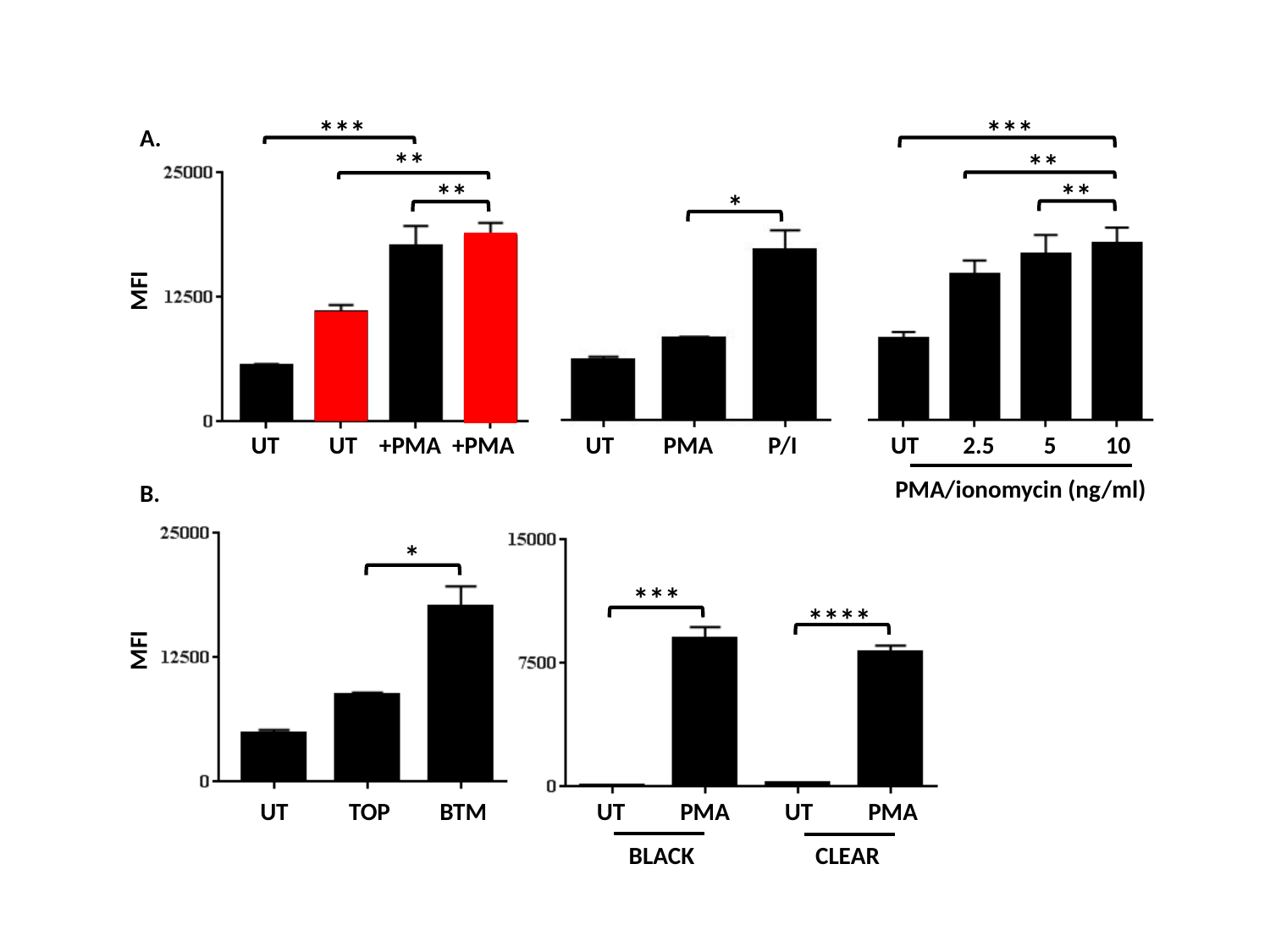

***
***
A.
B.
**
**
**
**
*
MFI
 UT UT +PMA +PMA UT PMA P/I UT 2.5 5 10
 PMA/ionomycin (ng/ml)
*
***
****
MFI
 UT TOP BTM UT PMA UT PMA
 BLACK CLEAR

Supplement: S2 Fig — (A) 5x10^5^ HL-60 human neutrophil-like cells were stimulated or not with PMA/ionomycin for 30 minutes. During the final 5 minutes of stimulation, the media was supplemented with either 1μg/ml (black bars) or 2μg/ml (red bars) DHR-123 and then analyzed for fluorescence at 488nm indicative of the presence of reactive oxygen species. Additional graphs depict comparisons between initial stimulations of PMA or PMA/ionomycin (P/I) and 2.5, 5 or 10ng/ml P/I. (B) Comparisons of optimally stimulated HL-60 cells read from the top or bottom and in black and clear-bottom well plates. * (p<0.05), ** (p<0.01), *** (p<0.001) and **** (p<0.0001). (PPTX) [file pone.0146105.s002.pptx]

## Slide 1
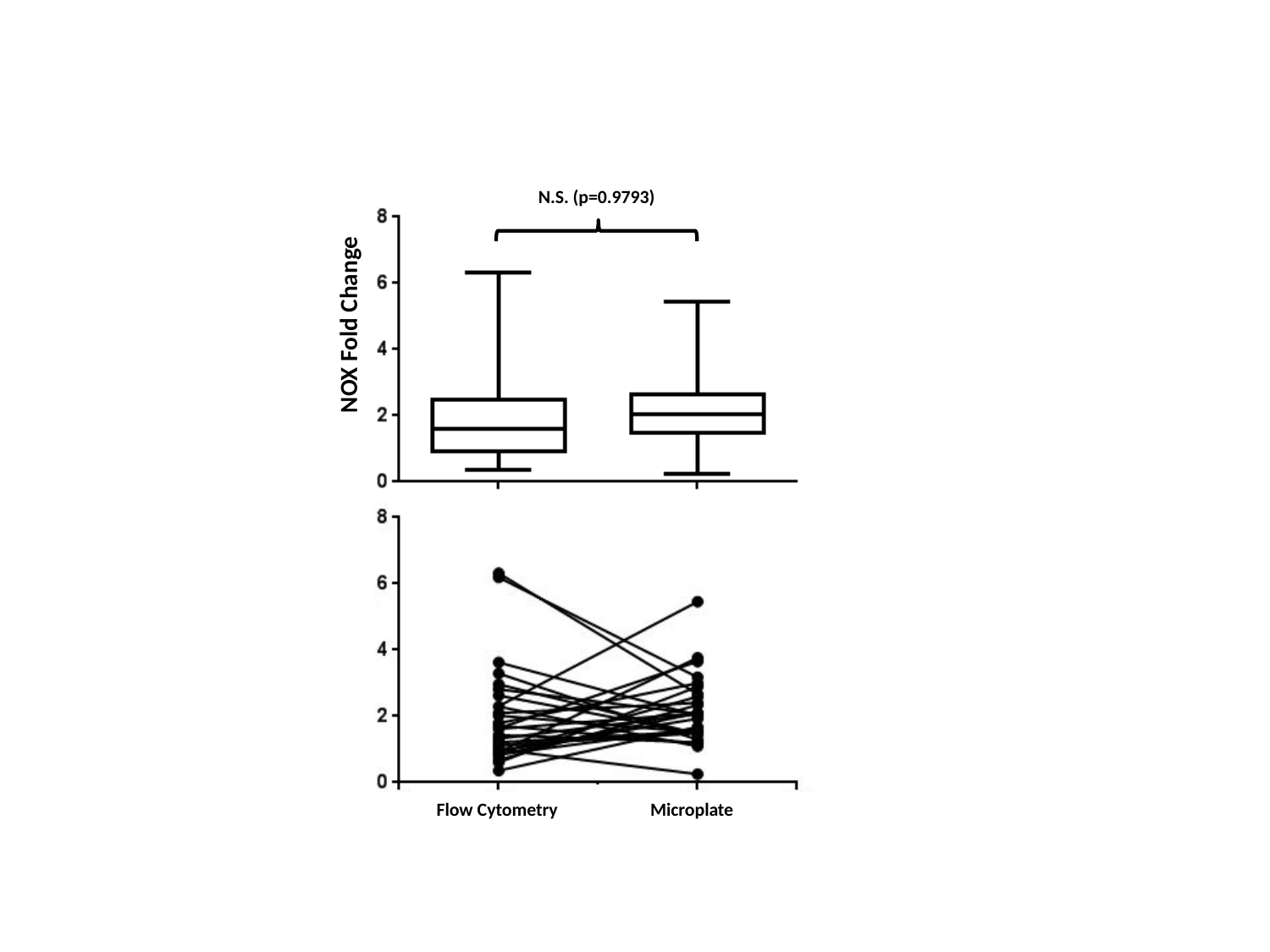

N.S. (p=0.9793)
NOX Fold Change
 Flow Cytometry Microplate

Supplement: S3 Fig — NOX capacity was measured by flow cytometer and microplate reader in paired samples of Rhesus Macaque whole blood and directly compared. There were no significant differences in assay performance, sensitivity or variability amongst individual samples (p = 0.9793). (PPTX) [file pone.0146105.s003.pptx]
